# Outcomes of odontoid fractures with associated cardiac arrest: retrospective bi-center case series and systematic literature review

**DOI:** 10.1186/s13049-024-01277-z

**Published:** 2024-10-29

**Authors:** S. F. Schaible, S. Häckel, N. Rutsch, F. C. Aregger, S. F. Bigdon, V. Schoenborn, I. Broger, C. E. Albers, C. Tinner

**Affiliations:** 1 Department of Orthopaedic Surgery and Traumatology, University Hospital Bern, Inselspital, University of Bern, Freiburgstrasse 20, Bern, CH-3010 Switzerland; 2 Department of Orthopaedic Surgery and Traumatology, Cantonal Hospital Graubuenden, Loestrasse 99, Chur, CH-7000 Switzerland; 3 Graduate School for Health Sciences, University of Bern, Bern, Switzerland

**Keywords:** Cervical injuries, Spinal injuries, Heart arrest, Resuscitation, Review

## Abstract

**Background:**

Odontoid fractures from high-energy trauma are associated with significant morbidity and mortality, including spinal cord injury, neurological damage, and cardiac arrest. The literature on odontoid fractures leading to cardiac arrest is limited to isolated case reports. This study aims to conduct a retrospective bi-center case series and a systematic review of existing literature.

**Methods:**

We conducted a retrospective bi-center case series on patients with odontoid fractures from high-energy trauma who experienced post-traumatic cardiac arrest with return of spontaneous circulation (ROSC) after CPR from two Level 1 Trauma Centers (2008–2024). The primary outcome was in-hospital mortality; secondary outcomes included epidemiological, pre-hospital, and in-hospital data, and CT and MRI findings. Additionally, we performed a systematic literature review to summarize existing evidence.

**Results:**

The study included 25 patients (mean age 71.1 ± 12.3 years, SD; 8 females). The mortality rate was 92% (23 patients). Median downtime before CPR was 5.0 min (IQR: 7.0), with CPR lasting 17.0 min (IQR: 13.0), primarily initiated by professionals (60%). All patients were quadriplegic. Type II Anderson d’Alonzo fractures were most common (88%), with all patients showing myelopathy on MRI. Only three patients (12%) underwent surgical intervention due to favorable prognosis. Our literature review identified seven case reports, with two patients surviving and one achieving full recovery.

**Conclusions:**

In this case series, patients experiencing cardiac arrest after odontoid fractures exhibited high mortality rates despite comprehensive management at Level 1 trauma centers. Survivors faced significant and enduring morbidity.

## Background

Odontoid fractures constitute a significant proportion of acute cervical spine injuries, accounting for 9–18% of cases. These fractures predominantly affect patients aged 65 years and older, typically resulting from low-energy trauma [1–3]. However, they can also occur in younger patients following high-energy trauma, potentially associated with neurological deficits. These fractures significantly impair quality of life and burden patients and healthcare systems. Overall, mortality rates reach 14% within 30 days and 44% within two years, with neurological injury incidence between 2% and 27% [4]. While spinal cord injury (SCI) rarely accompanies low-energy falls, high-energy spine trauma can cause SCI through traction, contusion, compression, or ischemia. In severe cases, acute high-level SCI may trigger cardiac arrest by disrupting the sympathetic nervous system [5–9].

Disrupted preganglionic sympathetic interneurons leads to parasympathetic dominance ending in bradyarrhythmias and atrioventricular blocks [10]. In these severe cases of odontoid fractures with subsequent cardiac arrest, immediate intervention requires cardiopulmonary resuscitation (CPR) as a life-saving measure, followed by comprehensive management of polytrauma, including damage control surgery, and intensive or intermediate care based on patient condition and diagnostic imaging findings [11–13]. Persistent spinal cord compression necessitates prompt decompression, with subsequent operative stabilization often required for managing the typically unstable fracture patterns of odontoid fractures associated with SCI [14–21]. However, in cases of severe spinal cord damage, the prognosis for neurological recovery is poor [17, 18].

While there is substantial data on the treatment of isolated odontoid fractures and on cardiac arrest independently, the literature addressing the unique combination of odontoid fractures causing cardiac arrest due to high spinal cord or medulla oblongata injuries is notably sparse. The existing studies are primarily isolated case reports, which often document patient mortality within days of the injury.

To address this gap, in this study, we present a bi-center case series aimed at exploring the treatments and outcomes of odontoid fractures followed by cardiac arrest with return of spontaneous circulation (ROSC), with the following objectives: first, mortality and descriptive analysis of pre- and in-hospital treatment; second, a systematic review of literature on comparable scenarios; and third, a comparison of our case series with the literature findings.

## Methods

### Cohort definition and outcomes

After obtaining ethics approval from local ethics committees (Kantonale Ethikkommission Bern [KEK]); BASEC-Nr: Req-2023-00618), we performed a retrospective review of the databases of two Swiss level 1 trauma centers between 2008 and 2024, University Hospital Bern (Inselspital) and Cantonal Hospital Graubuenden Chur. We included all patients who experienced a high-energy trauma according to a recently published definition [22], who were resuscitated at the accident scene, who achieved ROSC, and who were diagnosed in the emergency department with an odontoid fracture that was determined to be the primary cause for cardiac arrest necessitating the resuscitation. Exclusion criteria were absence of an odontoid fracture, failure to achieve ROSC, incomplete data, patients under 18 years of age, and lack of general consent. Patient data were handled with strict confidentiality and in compliance with data protection regulations. Only essential information such as age, gender, and diagnosis were extracted from the database in a pseudo-anonymized fashion to ensure patient privacy.

Our primary outcome was patients’ in-hospital mortality, defined as death occurring during the hospitalization period. Secondary outcomes included pre-hospital and in-hospital treatment. To collect pre-hospital and in-hospital data, we reviewed reports from the evacuation team, emergency department, and intensive care unit (ICU). Pre-hospital treatment outcomes included the duration of cardiac arrest, duration, and providers of CPR (categorized as layperson bystanders, professionals, or both), administered dose of adrenaline, and time to ROSC. In-hospital outcomes encompassed return to consciousness, best neurological status in the ICU, treatment of the odontoid fracture (surgery, conservative, or best supportive care), time to death, time to ICU discharge, and time to hospital discharge. Additionally, we recorded injury mechanisms and epidemiological data from hospital records.

We retrospectively classified the fractures identified on the initial whole-body computed tomography (CT) scans, assessing the degree of fracture displacement. If available, magnetic resonance imaging (MRI) studies were evaluated for the presence of myelopathy and stenosis with/without persisting compression of the spinal cord. Additionally, we used the Brain and Spinal Injury Center (BASIC) score, which evaluates acute traumatic spinal cord injury by grading the axial extent of intramedullary signal abnormalities on T2-weighted MRI on a scale from one to four [23].

### Systematic literature review

On April 1, 2024, we conducted a systematic literature review following the current Preferred Reporting Items for Systematic Reviews and Meta-Analyses (PRISMA) guidelines [24]. Our search included the PubMed, Google Scholar, and Medline databases, using the terms (“odontoid fracture” AND “cardiac arrest”) and (“odontoid fracture” AND “arrest”) applied to all fields. The inclusion criteria were studies reporting cases of odontoid fractures associated with cardiac arrest before or during emergency treatment, English publications, and original research, case reports, or case series. We excluded studies where cardiac arrest was unrelated to odontoid fractures, occurred later during hospitalization, non-English publications, and those without full-text availability. To filter the initial search results, we used Rayyan AI [25], an automated screening tool, to exclude duplicates and irrelevant abstracts based on titles. Two independent reviewers then manually screened the remaining abstracts using the inclusion and exclusion criteria, identifying eligible articles. Their full texts were reviewed, and discrepancies resolved through consensus. Data from the selected articles were extracted and systematically analyzed, focusing on the same outcomes as described for our own cohort, as well as epidemiological data and study designs.

### Statistical methods

Descriptive statistics were employed to present the study outcome data. Data collection was conducted using password-protected software, and analysis was performed using R (R Foundation for Statistical Computing, Vienna, Austria). A Shapiro-Wilk test was conducted to assess for normal distribution. Continuous data are presented as mean ± standard deviation (SD) if normally distributed, or as median and interquartile range (IQR) if not.

## Results

**Fig. 1:**
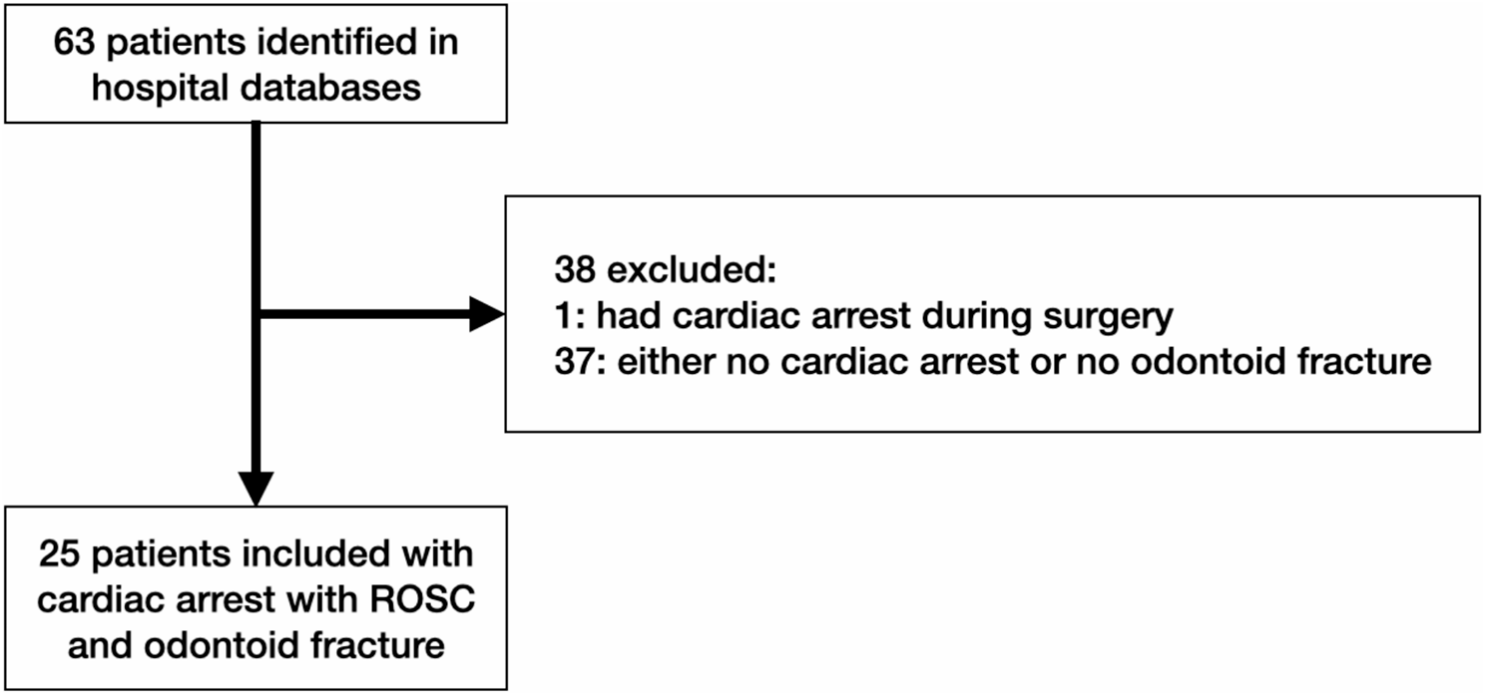
Patient selection flow chart

We included 25 patients who suffered a high energy odontoid fracture that were identified as the primary cause for immediate cardiac arrest, and that were followed by ROSC after CPR (Fig. 1; Table 1).

**Table 1:** Cohort demographics, injury characteristics, treatment, and primary outcome

Case	Age	Sex	Comorbidities	Charlson Comorbidity Index	Trauma mechanism	Anderson d’Alonzo Classification	AO Spine Upper Cervical (Primary Injury)	Treatment	Discharge/Death	Cause of Death
1	71	M	Moderate to severe CKD^1^	5	Staircase fall	II	II C; M1-3	Cardiorespiratory support	Death	Brain death, terminal prognosis
2	73	W	-	3	Staircase fall	II	III A; M1-3	Ventral Dens axis screw	Discharge	N/A
3	42	M	-	0	Bike accident	III	III A; M1,2	Cardiorespiratory support	Death	Brain death, terminal prognosis
4	69	W	MI^2^, uncomplicated diabetes mellitus	4	Unknown	II	III A; M1-3	Cardiorespiratory support	Death	Brain death, terminal prognosis
5	77	W	Moderate to severe CKD^1^	5	Unknown	II	III A; M1-3	Cardiorespiratory support	Death	Brain death, terminal prognosis
6	89	M	TIA^3^	5	Unknown	II	III C; M1-3	Cardiorespiratory support	Death	High SCI^4^ with respiratory insufficiency, terminal prognosis
7	38	M	-	0	Bike accident	III	III A; M1,2	Cardiorespiratory support	Death	Brain death, terminal prognosis
8	64	M	-	0	Bike accident	II	III A; M1,2	Decompression and open posterior stabilization Th2-Th7	Death	Brain death, terminal prognosis
9	67	W	Peripheral vascular disease, uncomplicated diabetes mellitus	4	Unknown	II	II C; M1-3	Cardiorespiratory support	Death	Brain death, terminal prognosis
10	82	W	-	4	Unknown	II	III A; M1,2	Cardiorespiratory support	Death	High SCI with respiratory insufficiency, terminal prognosis
11	83	W	-	4	Staircase fall	II	III A, M1-3	Cardiorespiratory support	Death	Brain death, terminal prognosis
12	85	M	-	4	Fall while walking	II	II C; M1-3	Cardiorespiratory support	Death	High SCI with respiratory insufficiency, terminal prognosis
13	56	M	-	1	Bike accident (polytrauma^a^)	II	III A; M1-3	Cardiorespiratory support	Death	Brain death, terminal prognosis
14	68	M	-	2	Bike accident	II	III A, M1-3	Cardiorespiratory support	Death	Brain death, terminal prognosis
15	80	W	CHF^5^, peripheral vascular disease, TIA^3^, COPD^6^	9	Staircase fall (polytrauma)	II	III A, M1-3	Cardiorespiratory support	Death	High SCI with respiratory insufficiency, terminal prognosis
16	74	M	-	3	Bike accident	II	III A; M1-4	Cardiorespiratory support	Death	Brain death, terminal prognosis
17	73	M	-	3	Tractor accident (polytrauma)	II	III A; M1-3	Cardiorespiratory support	Death	Brain death, terminal prognosis
18	64	M	Localized solid tumor	3	Bike accident	II	III A; M1-3	Cardiorespiratory support	Death	High SCI with respiratory insufficiency, terminal prognosis
19	68	M	-	2	Ski accident	III	III A, M1-3	Screw fixation of the dens (cervical vertebra), MIPO (Minimally Invasive Plate Osteosynthesis) of the tibia	Discharge	N/A
20	64	M	-	2	Ski accident	II	III A; M1-3	Cardiorespiratory support	Death	High SCI with respiratory insufficiency, terminal prognosis
21	74	M	-	3	Ski accident	II	III C; M1-3	Cardiorespiratory support	Death	High SCI with respiratory insufficiency, terminal prognosis
22	76	M	-	3	Bike accident	II	III A, M1-3	Cardiorespiratory support	Death	High SCI with respiratory insufficiency, terminal prognosis
23	77	W	-	3	Bike accident	II	III A; M1-3	Cardiorespiratory support	Death	High SCI with respiratory insufficiency, terminal prognosis
24	76	M	-	3	Bike accident	II	III A, M1-3	Cardiorespiratory Support	Death	High SCI with respiratory insufficiency, terminal prognosis
25	88	M	-	4	Staircase fall	II	III A, M1-3	Cardiorespiratory Support	Death	High SCI with respiratory insufficiency, terminal prognosis

^1^CKD: chronic kidney disease; ^2^MI: myocardial infarction; ^3^TIA: transient ischemic attack; ^4^SCI: spinal cord injury; ^5^CHF: congestive heart failure; ^6^COPD: chronic obstructive pulmonary disorder

^a^polytrauma indicates an injury severity score (ISS) > 15 [26]

The mean age was 71.1 years ± 12.3 (SD), and eight patients (32%) were female. The dominant trauma mechanisms were bike accidents in ten cases (40%). Three patients (12%) had a polytrauma with an injury severity score (ISS) of 15 or greater [26].

CPR was administered to all patients. In 22 cases (88%), CPR was initiated at the site of the accident. The initiation of CPR was consistently prompt, with a median downtime of just five minutes, followed by comprehensive care at Level 1 trauma centers in all cases. CPR was initiated by professionals in 15 cases (60%), and by layperson bystanders in 10 cases (40%). The median time to professional CPR was 15 min. Seventeen patients (68%) of patients received adrenalin at the accident site. The mean duration of CPR was 17 min; 25 patients (100%) achieved ROSC.

CT scans revealed Type II odontoid fractures according to Anderson d’Alonzo as the predominant fracture pattern (88%), and Type A (60%) according to the AO Spine Upper Cervical Classification. Additionally, four patients (16%) presented with atlantoaxial (C1-C2) dislocation. Twenty-one patients (84%) underwent MRI after CT. Radiographic signs of cervical myelopathy were present in all 21 patients who received an MRI of the cervical spine (84% of all patients). Ongoing compression of the spinal cord/medulla oblongata was present in one patient (4%). Every patient had BASIC Score of three or higher, indicating the absence of residual normal-appearing white matter (Table 2).

**Table 2:** Cohort characteristics pre-treatment (descriptive statistics)

Epidemiology	Total (*n*=25)
Age, mean ± SD^1^	71.1 ± 12.3
Females, n (%)	8 (32)
Trauma mechanism, n (%)	
Bicycle	10 (40)
Staircase fall	5 (20)
Ski	3 (12)
Other/unknown	7 (28)
Pre-hospital CPR^2^, n (%)	22 (88)
CPR performer, n (%)	
Professionals	15 (60)
Lay bystanders	5 (20)
Both	5 (20)
Time to start of CPR, median (IQR^3^, range) in minutes	5 (7, 0-30)
Time to professional CPR, median (IQR, range), in minutes	15 (12, 3-85)
Time to ROSC, median (IQR, range), in minutes	17 (13, 2-30)
Adrenalin before ROSC^4^, n (%) Dosage, median (IQR, range)	17 (68) 1.5, (1, 1-7)
GCS^5^ after ROSC n (%)	
≤7	22 (88)
8-10	3 (12)
**Fracture classification systems (CT**^**6**^)	**Total (n=25)**
Anderson d’Alonzo Type, n (%)	
I:	0 (0)
II:	22 (88)
III:	3 (12)
AO Spine (Primary Injury)	
Upper Cervical Classification, n (%)	
Type A	20 (80)
Type B	0 (0)
Type C	5 (20)
Atlantoaxial (C1-C2) dislocation, n (%)	5 (20)
Displacement, median (IQR, range), in millimeters	3.5 (3.7, 0-13)
Associated C1 fractures, n (%)	6 (24)
Burst Anterior arch	2 (8) 2 (8)
Posterior arch	2 (8)
**MRI** ^7^ **findings**	**Total (n=21)**
Myelopathy, n (%)	21 (100)
Myelocompression, n (%)	1 (5)
BASIC^8^ Score, n (%)	
3	3 (14)
4	18 (86)

^1^standard deviation, ^2^cardiopulmonary resuscitation, ^3^interquartile range, ^4^return of spontaneous circulation, ^5^Glasgow coma scale, ^6^computed tomography, ^7^magnetic resonance tomography, ^8^Brain and Spinal Injury Center score

**Fig. 2:**
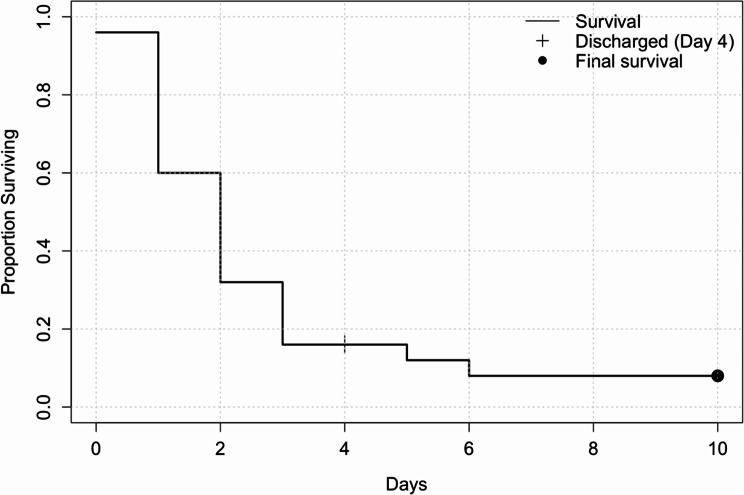
Survival plot

In total, only two patients (8%) survived, leading to a mortality rate of 92% (Fig. 2) (Table 3). All patients who died were treated on the ICU, and supportive treatment was ceased after interdisciplinary evaluation either due to high SCI with respiratory insufficiency (nine patients) or diagnosed brain death due to either hypoxia or head trauma (eleven patients). If contactable, patients were involved in this decision-making.

**Fig. 3:**
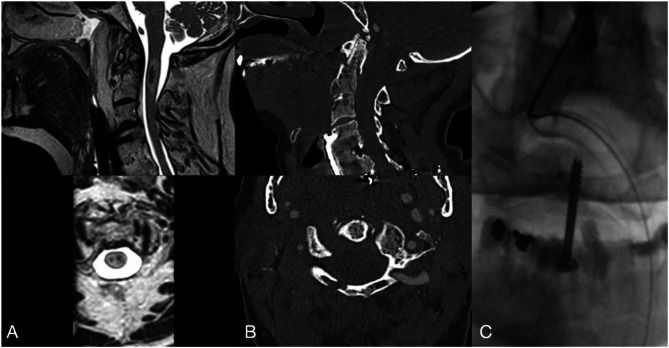
Case example patient number 2. 73 y/o hospitalized after a domestic fall from a staircase A top: sagittal and bottom: axial T2-weighted MRI imaging showing myelopathy (BASIC grade 4) without stenosis; B top: sagittal and bottom: axial CT imaging: showing posterior arch disruption of the atlas in an Anderson d’Alonzo type II (AO Type II B; M1-3) odontoid fracture with 3 mm displacement; C: intraoperative ap fluoroscopy of a ventral Dens axis screw; the patient regained consciousness on the ICU, was transferred to another ICU after two days, and survived

Only one patient (patient 2) regained consciousness on the ICU. Three patients (patients 2, 8, and 19; 12%) had sufficiently favorable prognosis due to neurological and cardiorespiratory improvement and proceeded to surgical fixation. Two of these three patients ultimately survived: The first survivor (patient 2) underwent odontoid screw fixation for an Anderson d’Alonzo type II (AO type II B; M1-3) fracture (Fig. 3). The second survivor (patient 19) with an Anderson d’Alonzo type III (AO type III A; M1-3) fracture was treated with an odontoid screw fixation. The third patient (patient 8), presenting with an Anderson d’Alonzo type II (AO type III A; M1-3) fracture, was treated conservatively for the odontoid fracture, but underwent decompression and open posterior stabilization from Th2 to Th7 due to an additional C-Type injury at Th4/5. He ultimately succumbed to his injuries.

**Table 3:** In-hospital outcomes

In-hospital Outcomes	Total (*n* = 25)
Mortality, n (%)	23 (92)
Time to Death, mean ± SD^1^ in days	2.0 (1.4)
Return to consciousness on ICU^2^, n (%)	1 (4)
Best Status on ICU^2^, n (%)	
GCS^3^ 15, spontaneous breathing, tetraplegic	1 (4)
GCS^3^ 11–14	0 (0)
GCS^3^ 8–10, contactable with eyes, no spontaneous breathing,	3 (12)
tetraplegic	
GCS^3^ 3, no spontaneous breathing, tetraplegic	21 (84)
Surgical treatment, n (%)	3 (12)
Time to Discharge from ICU^2^ in days	
Patient 2:	4
Patient 19:	4

^1^standard deviation, ^2^intensive care unit, ^3^Glasgow Coma Scale

### Systematic literature review

**Fig. 4:**
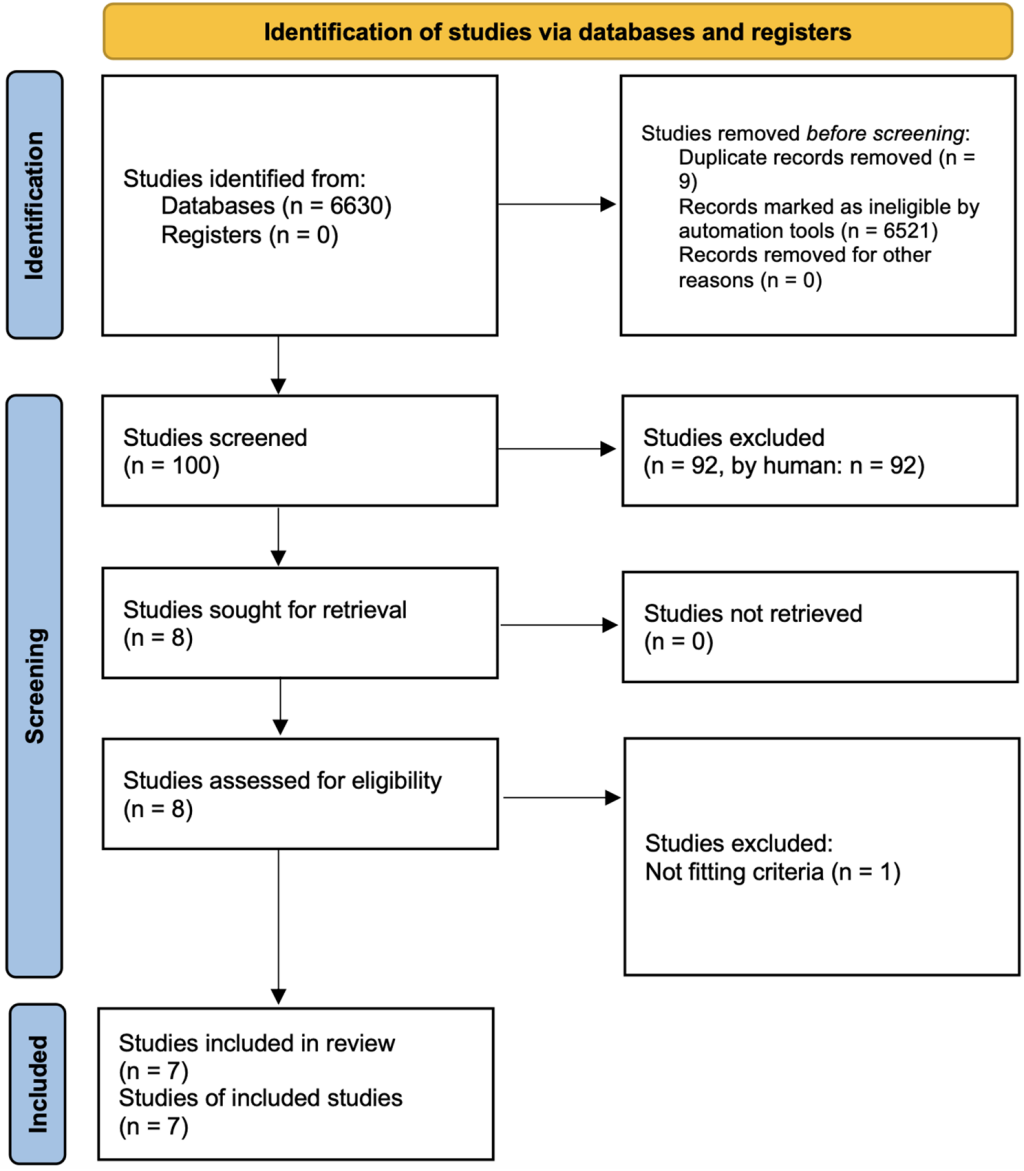
PRISMA 2020 flow diagram for systematic review which included searches of databases and registers only

In our systematic literature review, the initial search identified 6,630 articles. After screening and applying the inclusion criteria, we narrowed the selection to seven case reports that addressed patients with odontoid fractures associated with cardiopulmonary arrest [10, 27–32] (Fig. 4). These cases involve patients ranging in age from 20 to 68 years, with a mean age of 57 years. The mechanism of injury included car crashes (3 cases), domestic falls or collapses (2 cases), a bike accident (1 case), and a fall in the street (1 case). The odontoid fractures were predominantly Anderson-d’Alonzo type II fractures (4 cases). Two patients underwent surgical treatment. Of those, one patient underwent Glisson’s traction [29], and one patient received posterior fixation of C1-C2 [30]. Both patients survived. The first patient was weaned off the respirator after four months and became independent after six months [29]. For the second patient, no mid- and long-term follow-up was reported [30]. The remaining five patients succumbed to their injuries shortly after the incident [10, 27, 28, 31, 32] (Table 4).

**Table 4:** Published cases of odontoid fractures associated with cardiac arrest

Author (Year)	Patient	Trauma	Down Time	CPR	ROSC	Fracture	Myelopathy	Myelocompression	Neurological Deficit	Treatment	Return of Consciousness	Outcome
Kubokura [29]	20 y/o female	Car accident	Six minutes	Professional	Yes	Fracture of odontoid process Anterior with atlantoaxial dislocation	Not specified	Not specified	Quadriplegia	Traction by Glisson’s method	Five days	Weaned off the respirator after four months, independent after six months
Graziano [28]	62 y/o female	Car accident	Not specified	Professional	Yes	Anderson-d’Alonzo type II odontoid fracture with significant distraction but without significant subluxation	Not specified	Not specified	fixed, dilated pupils, no response to deep pain, absence of brainstem reflexes, including cold caloric stimulation	Cardiorespitatory support	No	full cardiac arrest within 2 h of arrival to hospital, died after unsuccessful CPR
Powell [32]	59 y/o male	Domestic collapse of unknown etiology	Not specified	Professional	Yes	Fracture of the odontoid process fracture with 4 mm posterior dislocation	Not specified	Not specified	Apnea, clinical signs consistent with a complete spinal cord injury below C2	Cardiorespiratory support on ICU	No	Death after three days
Bowers [10]	58 y/o female	Domestic fall	None	Bystander CPR and medical reanimation	Yes	Anderson-d’Alonzo type III odontoid fracture; Jefferson-type fracture of the atlas	Yes	No	Apnea, quadriplegia with complete paralysis below the neck	Cardiorespiratory support on ICU	No	Death (unspecified)
Pérez-Bovet [31]	68 y/o male	Car accident	None	Professional (in-hospital arrest)	No	Anderson-d’Alonzo type IIA odontoid fracture (base fracture with anterior luxation)	Not specified	Not specified	Initially without deficit	CPR	No	Death during CPR
Barber Ansón [27]	67 y/o male	Bike accident	Not specified	Not specified	Yes, after 34 min	Anderson-d’Alonzo type II odontoid fracture	Yes	No	Apnea, quadriplegia with complete paralysis below the neck	Cardiorespiratory support on ICU	No	Death after six days
Maeda [30]	62 y/o male	Fall in the street	None	Bystander CPR and medical reanimation	Yes, after 3 min	Anderson-d’Alonzo type II odontoid fracture	Yes	Yes	Difficulty in movement of arms and legs, hypoesthesia (ASIA D)	Posterior fixation of C1-C2	Yes	Discharge (unspecified)

## Discussion

Our retrospective analysis of 25 cases from two Level 1 Trauma Centers presents the largest case series to date of odontoid fractures with concurrent cardiac arrest and ROSC after CPR. Despite successful pre-hospital resuscitation, the mortality rate was 92% after mean of 2.0 ± 1.4) days.

The global incidence of out-of-hospital cardiac arrest is estimated at 55 cases per 100,000 person-years, with survival rates varying widely due to differences in emergency medical response and public health infrastructure. Typically, shorter downtimes, such as the median of 5 min in our cohort, result in much higher survival rates of up to 60% when the downtime is 0–10 min, and still in the double digits even when exceeding 20 min, as shown by larger series [33, 34].

Odontoid fractures significantly elevate mortality risks due to various factors, including neurological damage leading to respiratory arrest and complications from immobilization, such as cardiovascular, respiratory, and septic issues, resulting in nearly 15% mortality within 30 days [35, 36]. Our findings reveal that despite rapid intervention and advanced care, the prognosis for patients suffering from odontoid fractures with concurrent cardiac arrest remains grim: with survival and consciousness recovery rates below 10%, our study echoes observations from earlier case reports that similarly document minimal survival in such scenarios, with only two cases reporting survival [29, 30]. This underscores the severe impact of the combined scenario of cardiac arrest with odontoid fracture and spinal cord injury, indicating a compounded risk that significantly lowers survival prospects. Conversely, another study analyzing out-of-hospital cardiac arrests found that the optimal cut-off for favorable neurological outcomes is 12 min of CPR, which is shorter than the mean duration reported in our cohort [37]. This suggests that considerable damage has already occurred by the time ROSC is achieved.

As highlighted in earlier reports, initial cardiac arrests can obscure severe spinal injuries [27]. Odontoid injuries are primarily of two types: one involves potentially fatal complete atlanto-axial (C1-C2) displacement, and the other features slight displacement with complete rupture of the capsular structure, leading to significant instability [28]. Recognizing the latter is crucial for initiating immobilization to prevent further neurological damage.

The atlanto-axial vertebrae region has a notably wide margin of safety within the spinal canal, allowing substantial fracture displacement without immediate contact between the odontoid fragment or the posterior ring of the first cervical vertebra and the spinal cord [38]. Acute and severe neurological impairments, such as tetraplegia accompanied by cardiac arrest, often indicate high-energy trauma involving at least temporary high-grade dislocation. However, even low-velocity accidents, like falls from standing height with whiplash mechanisms, can lead to odontoid fractures with neurological impairment and cardiac arrest [39]. In our series, one patient (4%) and two out of seven cases (29%) in our systematic review experienced such trauma [10, 30]. We support the hypothesis that in these low-energy scenarios, particularly among elderly patients with reduced cervical spine flexibility, the odontoid process can endure high localized forces. This can result in transient dislocation and spinal cord damage despite the low-energy nature of the incident [35, 40]. It is crucial to consider this mechanism in cases of cardiac arrests following falls, even in seemingly low-energy accidents, to initiate further diagnostics and spinal immobilization promptly.

Pathophysiologically, neurogenic shock induced by autonomic dysfunction from high cervical spinal cord injuries typically leads to catastrophic outcomes [41]. This condition primarily disrupts preganglionic sympathetic interneurons that originate in the hypothalamus and exit the spinal cord between T1 and T6. The resultant shift towards parasympathetic dominance triggers severe bradyarrhythmias and atrioventricular blocks [10]. Our case series, which exclusively involved patients with cardiac arrest after odontoid fractures, appears to corroborate this pathophysiology. It is further supported by MRI findings showing severe signs of myelopathy in all patients, consistent with other reported cases [10, 30, 42]. Typically, neurological symptoms develop with 7–9 mm of lateral fracture displacement instability [35]. However, in our series, the mean displacement at the time of imaging was less, suggesting that severe dislocation may have occurred during the accident, evidenced by the high rates of myelopathy. Caregivers should consider the connection between odontoid fractures and cardiac arrest, even when trauma CT shows low dislocation.

Early suspicion of odontoid fractures, particularly in the elderly, is critical due to their nonspecific presentation and rapid deterioration, as demonstrated by a case where cardiac arrest occurred in the hospital [31]. These findings underscore the necessity of immediate and controlled spinal immobilization in unconscious patients, regardless of the trauma mechanism. Additionally, they highlight the importance of prompt CT imaging and the early involvement of neurosurgery, orthopedics, and critical care specialists to effectively coordinate care in suspected spinal trauma cases.

The use of MRI in previous case series has been inconsistent, with no clear guidelines on when to perform it. At our centers, the consensus is to conduct an MRI when spinal trauma is indicated on CT and the patient’s neurological status cannot be assessed, typically due to deep sedation, as was the case for all 25 patients in this series. This MRI is scheduled at an appropriate time when more critical issues have been stabilized. This approach is consistent with recommendations from other authors [43]. Additionally, an MRI is considered particularly prudent if neurological injury or ligamentous damage is suspected based on imaging findings or clinical history. The validity of this protocol is underscored by the 100% presence of myelopathy in our cases, indicating a high pretest probability that further supports the utility of this approach.

In treating odontoid fractures, the decision between surgical and conservative approaches depends on specific clinical criteria and patient characteristics: Surgical options, including anterior and posterior approaches, provide high rates of fracture stability and fracture union; notably, posterior instrumented fusion of C1-C2 also minimize complications such as dysphagia associated with anterior methods [4]. Conservative approaches are recommended for patients with less severe injuries or significant surgical risks [44]. In our series, two patients underwent successful screw fixation for a type II Anderson d’Alonzo fracture with 3 mm displacement and a type III fracture with 4 mm displacement, both without dislocation, but high risk of instability and non-union. Both had shown neurological improvement prior to surgery. This was a prerequisite for the chosen treatment.

Our findings have important clinical implications for managing patients with odontoid fractures associated with cardiac arrest, which can be summarized as follows:

First, in cases of trauma-associated cardiac arrest, clinicians should maintain a high index of suspicion for potential high cervical spinal cord injuries. Immediate spinal immobilization and prompt imaging with CT and MRI are essential to prevent further injury and to assess the extent of damage.

Regarding surgical intervention, there are two main pathways:

1. **No Persistent Spinal Cord Compression**: If imaging confirms an odontoid fracture with myelopathy but without ongoing spinal cord compression, immediate surgical decompression or stabilization may not be necessary. The primary focus should remain on effective cardiopulmonary resuscitation and stabilization of vital functions. For patients who achieve cardiopulmonary stabilization and show neurological improvement, timely surgical stabilization can be considered during hospitalization to prevent future instability or deformity.
2. **Persistent Spinal Cord Compression**: Conversely, if initial imaging reveals myelopathy with persistent spinal cord compression, immediate surgical intervention with spinal decompression and stabilization should be contemplated to offer the patient a chance for neurological recovery.

This patient-specific decision-making approach emphasizes the importance of prioritizing life-saving measures while tailoring surgical interventions based on individual neurological and physiological status.

Beyond focusing solely on odontoid fractures associated with cardiac arrest, comparing our findings with studies on cardiac arrest following severe neurological injuries like traumatic brain injury (TBI) offers further context. These cohorts consistently report low but notable survival rates and emphasize the critical importance of prompt resuscitation and continuous neurological evaluation to guide treatment decisions [45–47]. In TBI cases, interventions may include intracranial pressure monitoring or surgical craniectomy for decompression [47]. Similarly, for odontoid fractures with cardiac arrest, the initial focus should be on resuscitation and stabilization, with immediate surgical interventions, such as decompression and stabilization, reserved for rare instances of persistent myelocompression. Subsequent management should be tailored based on neurological assessments, considering surgical stabilization when there is potential for general and neurological improvement. Our findings align with this broader understanding, reinforcing that while survival rates are low, they are not zero. A systematic, patient-centered approach that prioritizes immediate life-saving measures and carefully evaluates the potential benefits of surgical interventions is essential in managing these complex cases.

The limitations of this study stem primarily from its design; it is retrospective in nature and includes a relatively small sample size coupled with a brief follow-up period. These factors significantly restrict the generalizability of our findings. However, it is worth noting that despite these limitations, this study represents the largest series to date addressing this rare and severe scenario as per our systematic literature review. The small sample size also prevented the execution of robust statistical analyses, highlighting the need for larger trials. Future research should aim to employ larger sample sizes to facilitate detailed statistical evaluations, such as linear regression, to identify factors associated with the outcomes studied.

## Conclusion

Cardiac arrest in conjunction with odontoid fractures is associated with a high mortality rate. In trauma-related cardiac arrest scenarios, it is critical to maintain a high index of suspicion for potential odontoid fractures. Beyond the immediate life-saving measures such as CPR, effective management includes the proper immobilization of the cervical spine and the timely and accurate use of diagnostic imaging to guide further treatment strategies.

## Data Availability

No datasets were generated or analysed during the current study.
